# High PRMT5 expression is associated with poor overall survival and tumor progression in bladder cancer

**DOI:** 10.18632/aging.103198

**Published:** 2020-05-11

**Authors:** Lei Tan, Kanghua Xiao, Yunlin Ye, Haitao Liang, Mingkun Chen, Junhang Luo, Zike Qin

**Affiliations:** 1 Department of Urology, The First Affiliated Hospital of Sun Yat-Sen University, Guangzhou, Guangdong, China; 2 State Key Laboratory of Oncology in South China, Sun Yat-Sen University Cancer Center, Guangzhou, China; 3 Department of Urology, Sun Yat-Sen University Cancer Center, Guangzhou, China; 4 Department of Urology, The Third Affiliated Hospital of Southern Medical University, Guangzhou, Guangdong, China

**Keywords:** bladder cancer, PRMT5, prognosis, cell cycle, mTOR

## Abstract

Arginine methyltransferase 5 (PRMT5) is involved in a variety of cancers. We used bioinformatics analysis to investigate PRMT5 overexpression in bladder urothelial cancer (BUC) and its clinical significance. We also conducted molecular biology experiments to investigate the effect of PRMT5 on the phenotype of BUC cells in vitro and in vivo. PRMT5 was found to be upregulated in BUC tissue in the Oncomine and The Cancer Genome Atlas databases. We validated the results from these databases in a cohort of BUC samples. Kaplan-Meier and Cox multivariate analyses demonstrated that PRMT5 upregulation is an independent prognostic risk factor for BUC. The in vitro and in vivo phenotypic experiments found that downregulated expression of PRMT5 in BUC cells inhibits BUC cell proliferation and aggression. In addition, gene set enrichment analysis demonstrated that PRMT5 knockdown leads to cell cycle G1/S arrest, deactivation of Akt, and mTOR phosphorylation in BUC cells. These results suggest that PRMT5 could be used as a potential molecular marker for BUC in the future.

## INTRODUCTION

Bladder urothelial cancer (BUC) is one of the most common genitourinary carcinomas in China [1]. It is also a significant cause of mortality in Western countries [2, 3]. BUC is categorized as muscle-invasive bladder cancer (MIBC) and non–muscle-invasive bladder cancer (NMIBC). Despite advances in surgical techniques and the development of novel drugs, NMIBC (stage Ta-1) with high morbidity tends to recur, whereas MIBC (stage T2-4) tends to metastasize within 2 years [3, 4]. Thus, it is important to identify the molecular mechanisms underlying tumorigenesis and metastasis of BUC so that new therapeutic targets and modalities can be developed to prevent and treat BUC [5]. BUC is thought to arise as a result of an accumulation of genetic alterations. At present, most studies on the etiology of BUC have focused on genetic transformation. Many oncogenes, such as C-myc [6], Bcl-2 [7], FGFR3 [8], and c-erbB-2 [9], have been found to be associated with BUC prognosis and progression [10]. However, the development of BUC is a complicated pathological process that requires further study.

Protein arginine methyltransferase 5 (PRMT5) is the predominant type II methyltransferase and is involved in numerous cellular and biological processes, such as Golgi homeostasis, nucleic acid metabolism, transcriptional regulation, cell cycle regulation, and ribosome biosynthesis [11, 12]. PRMT5 is a multifunctional gene with important functions in cells. Recent reports indicate that PRMT5 is overexpressed in a variety of human malignant tumors, suggesting that PRMT5 overexpression is an important high-risk factor and determinant of tumor properties. PRMT5 has been found to be upregulated in malignant tumors, such as gastric, lung, and prostate cancers [13–16]. One study demonstrated that overexpression and increased intranuclear accumulation of PRMT5 through methylation of Zn-finger protein were associated with a higher risk of defects in alternative splicing, leading to immortalized breast epithelial cells in humans [17]. The PRMT5 gene is a key regulator of amplification, migration, and metastasis of malignant tumors and participates in regulation of the cell cycle and autophagy. However, evidence is lacking regarding the relationship between PRMT5 expression and BUC.

Few diagnostic and prognostic genetic markers have been verified in BUC. Based on previous studies, this study was designed to detect PRMT5 gene expression in BUC and to assess its clinical significance. In addition, we studied the effect of PRMT5 on the proliferation and aggression of BUC.

## RESULTS

### PRMT5 is upregulated in BUC

**Figure 1:**
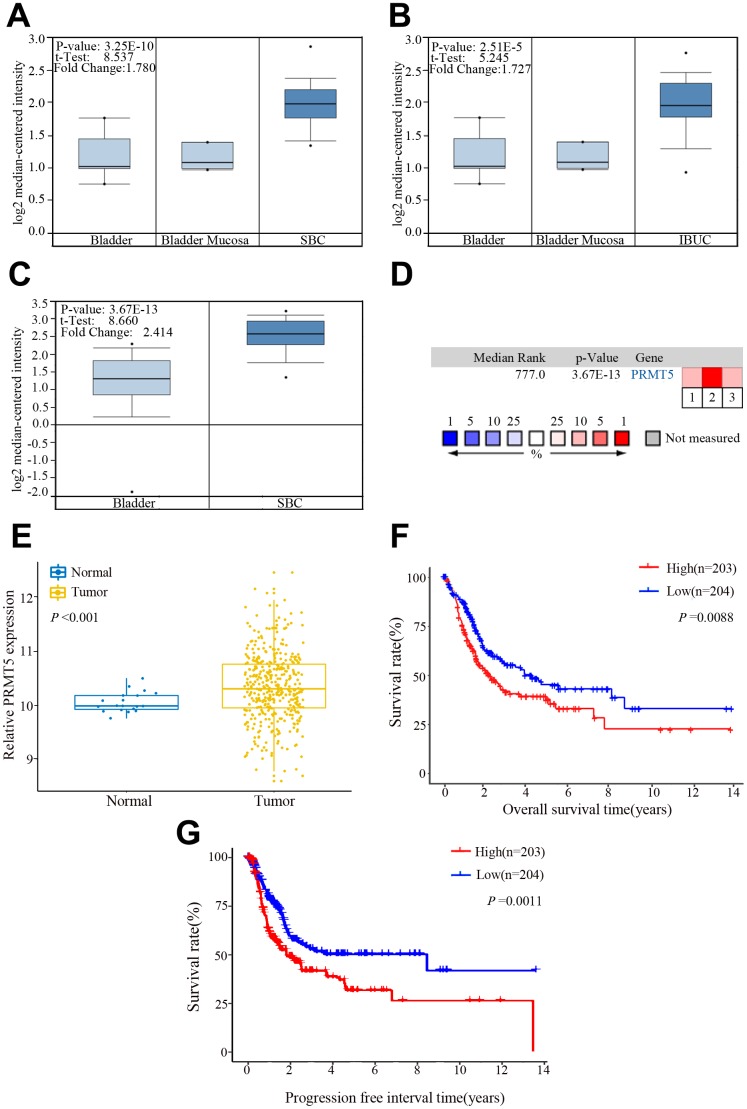
**PRMT5 expression in bladder cancer in the Oncomine and The Cancer Genome Atlas (TCGA) databases.** (**A–C**) Comparison of the PRMT5 expression levels in the Dyrskjot Bladder 3 and Sanchez-Carbayo Bladder 2 data sets of the Oncomine database. The threshold: *P* value: 1E-4, fold change: 1.5, gene rank: 10%. (**D**) A median-ranked analysis of the Dyrskjot Bladder 3 (1, 2) and Sanchez-Carbayo Bladder 2 (3) data sets from the Oncomine database. The colored squares revealed the median rank for PRMT5 across the three analyses (vs normal tissue). (**E**) Comparison of the PRMT5 expression level in bladder cancer and the normal tissue from the TCGA database. (**F**, **G**) Overall and progression-free survival times in bladder cancer patients with low versus high expression of PRMT5 assessed by Kaplan-Meier analysis from the TCGA cohorts. SBC: superficial bladder cancer, IBUC: infiltrating bladder urothelial carcinoma.

**Figure 2:**
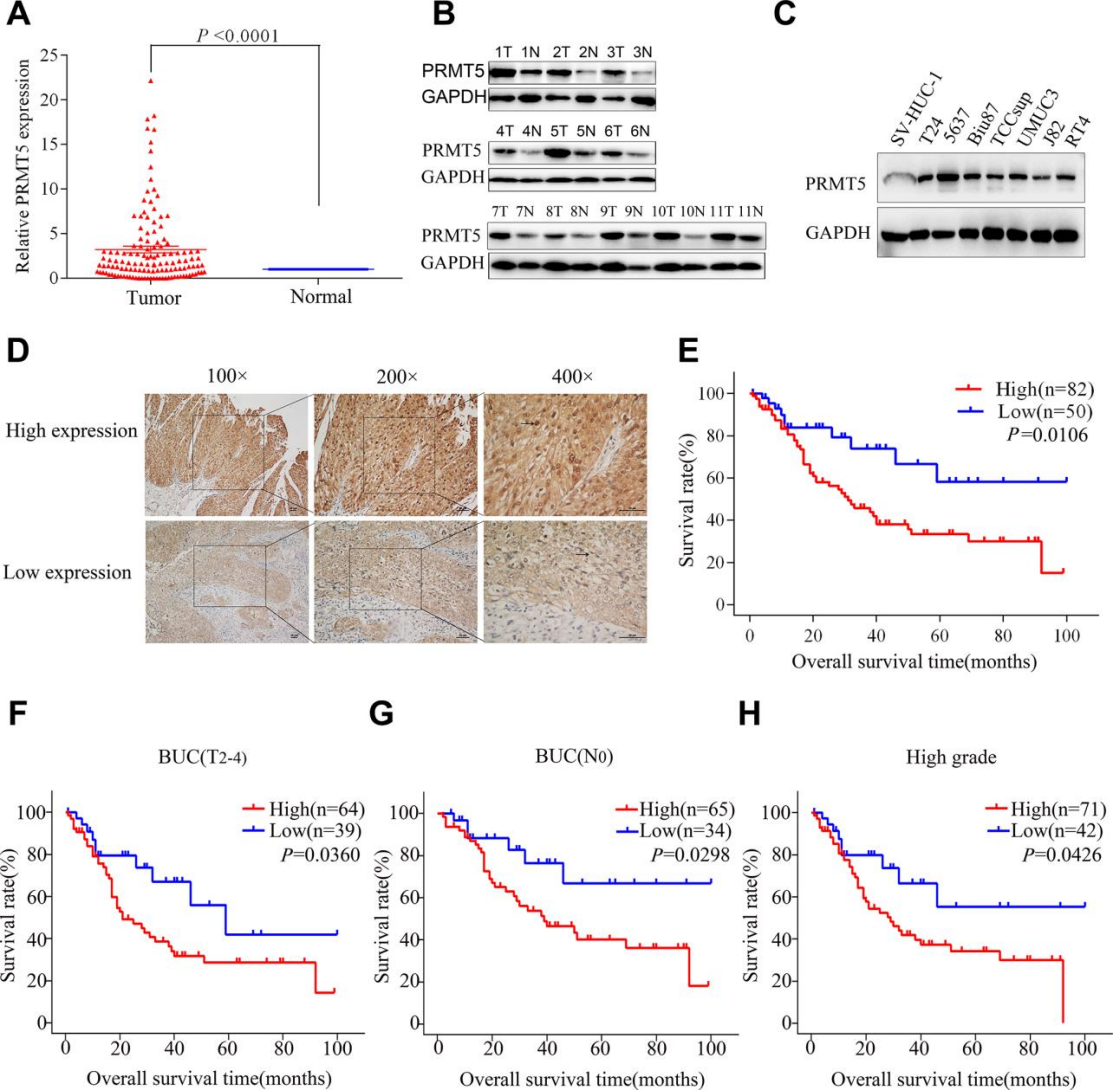
**PRMT5 was upregulated and demonstrated prognostic significance in bladder cancer.** (**A**) PRMT5 mRNA expression was significantly upregulated in bladder cancer tissue compared with that in adjacent normal tissues via qRT-PCR. (**B**) The PRMT5 protein level was upregulated in 11 pairs of bladder cancer tissues. (**C**) PRMT5 expression was upregulated in bladder cancer cell lines compared with immortalized human bladder epithelial SV-HUC-1 cells. (**D**) Representative images of immunohistochemistry of PRMT5 in bladder cancer tissues. (**E**) The Kaplan-Meier curve was applied to the survival analysis of bladder cancer patients with different PRMT5 expression levels from SYSUCC cohorts. (**F**–**H**) Positive correlation between overall survival and different PRMT5 expression levels from SYSUCC bladder cancer patients with muscle-invasive bladder cancer (**F**), absence of lymph node metastasis (**G**), and high-grade tumors (**H**). SYSUCC: Sun Yat-Sen University Cancer Center.

We performed bioinformatics analysis in three BUC data sets from the Oncomine database and found that PRMT5 was upregulated in superficial or infiltrating BUC tissue compared with nontumor bladder tissues (Figure 1A–1D). On further bioinformatics analysis using The Cancer Genome Atlas (TCGA) database, we confirmed that PRMT5 expression was higher in BUC tissue than in matched nonneoplastic bladder tissue (Figure 1E). In addition, in the TCGA database, high PRMT5 expression was associated with poor overall and progression-free survival in BUC patients (Figure 1F, 1G). To verify the database results, we determined the expression of PRMT5 mRNA in a cohort of 132 pairs of BUC and adjacent nontumor bladder tissues using quantitative and real-time polymerase chain reaction (qRT-PCR). We found that PRMT5 levels were significantly higher in BUC samples than in normal control samples (Figure 2A). In addition, PRMT5 protein was found to be overexpressed in BUC samples on western blotting assay and immunohistochemistry (Figure 2B–2D). Thus, we next focused on exploring the potential role of PRMT5 in BUC.

### PRMT5 upregulation is correlated with poor prognosis in BUC patients

Figure 2E shows that patients with high PRMT5 expression had a worse prognosis compared with patients with low expression (5-year overall survival rates, 33.3% *vs* 58.2%, respectively; *P* = 0.0106). The Kaplan-Meier curves also demonstrate poorer overall survival of patients with high PRMT5 expression, compared with those with low expression, with MIBC (T2-4) (*P* = 0.0360), absence of lymph node metastasis (*P* = 0.0298), and high-grade tumors (*P* = 0.0426; Figure 2F–2H). However, there was no significant association between PRMT5 expression and clinicopathologic parameters in BUC patients (Table 1). In addition, multivariate Cox proportional hazards regression analysis demonstrated PRMT5 upregulation to be an independent prognostic risk factor for worse survival of BUC patients (*P* = 0.012, Table 2). Thus, PRMT5 upregulation is associated with poor prognosis in BUC.

**Table 1:** The relationship between PRMT5 expression and clinicopathological characteristics in bladder cancer.

**Variables**	**No.**	**Expression of PRMT5 Level in BUC**		**χ^2^**	***P*value**
		**Low**	**High**		
Age (years)				0.158	0.691
<65	71	28	43		
≥65	61	22	39		
Gender				0.789	0.374
Male	108	39	69		
Female	24	11	13		
Tumor size (cm)				0.002	0.961
<3	69	26	43		
≥3	63	24	39		
T classification				2.580	0.630
Ta	6	3	3		
T1	22	7	15		
T2	25	8	17		
T3	44	20	24		
T4	34	11	23		
N classification				0.005	0.946
Negative	84	32	52		
Positive	48	18	30		
Grade				0.168	0.681
Low	19	8	11		
High/intermediate	113	42	71		

Statistically significant (*P* < 0.05). BUC: bladder urothelial cancer, T and N classification: TNM stage.

**Table 2:** Univariate and multivariate analyses of clinicopathological characteristics for survival in patients with bladder cancer.

**Variables**	**Univariate analysis *P* value**	**Multivariate analysis**	***P* value**
		**HR (95% CI)**	
Expression of PRMT5	**0.014**	**2.434 (1.215-4.876)**	**0.012**
Low			
High			
Age	0.081	1.542 (0.896-2.653)	0.118
<65 years			
≥65 years			
Gender	0.130		
Male			
Female			
Tumor size	0.169		
<3 cm			
≥3 cm			
T classification	<**0.001**	**1.576 (1.155-2.151)**	**0.004**
Ta			
T1			
T2			
T3			
T4			
N classification	**0.001**	1.482 (0.797-2.755)	0.213
Negative			
Positive			
Grade	0.097	1.209 (0.536-2.727)	0.674
Low			
High/intermediate			

HR: hazard ratio, CI: confidence interval. Bold values are statistically significant (*P* < 0.05).

### PRMT5 promotes proliferation, migration, and invasion of BUC cells

**Figure 3:**
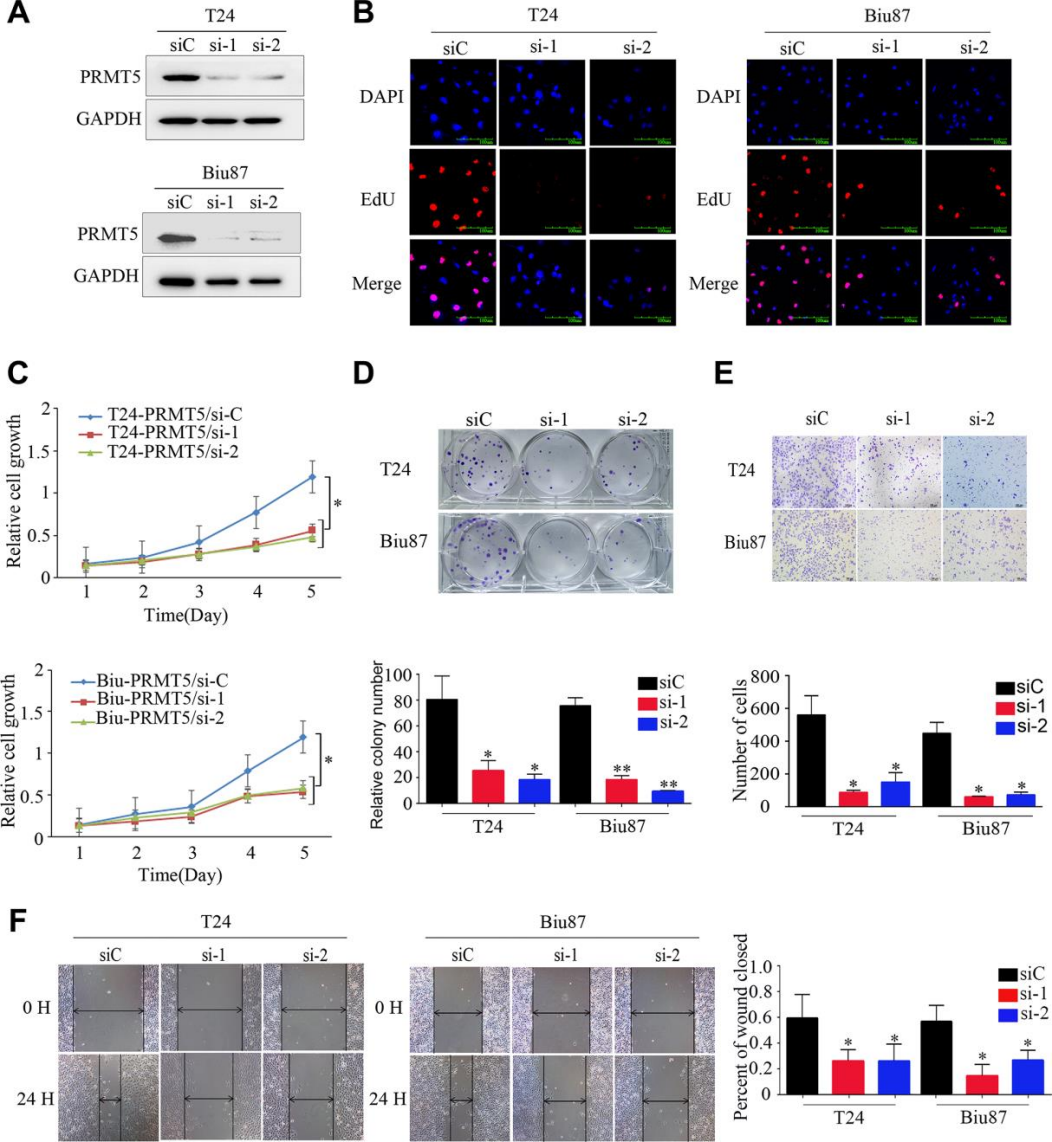
**Downregulated expression of PRMT5 inhibited the proliferation and aggressiveness of bladder cancer cells.** (**A**) PRMT5 was efficiently knocked down in T24-siRNA and Biu87-siRNA cells. (**B–D**) Cell proliferation was determined by EdU assay, CCK-8 assay, and colony formation assay of bladder cancer cells. (**E**, **F**) Cell invasion and migration capacities were determined using the transwell invasion assay and wound-healing assay for the indicated bladder cancer cells. Error bars show the standard error of the mean. siC: PRMT5-siRNA/negative control; si-1: PRMT5-siRNA/#1; si-2: PRMT5-siRNA/#2. *P < 0.05, **P < 0.01.

**Figure 4:**
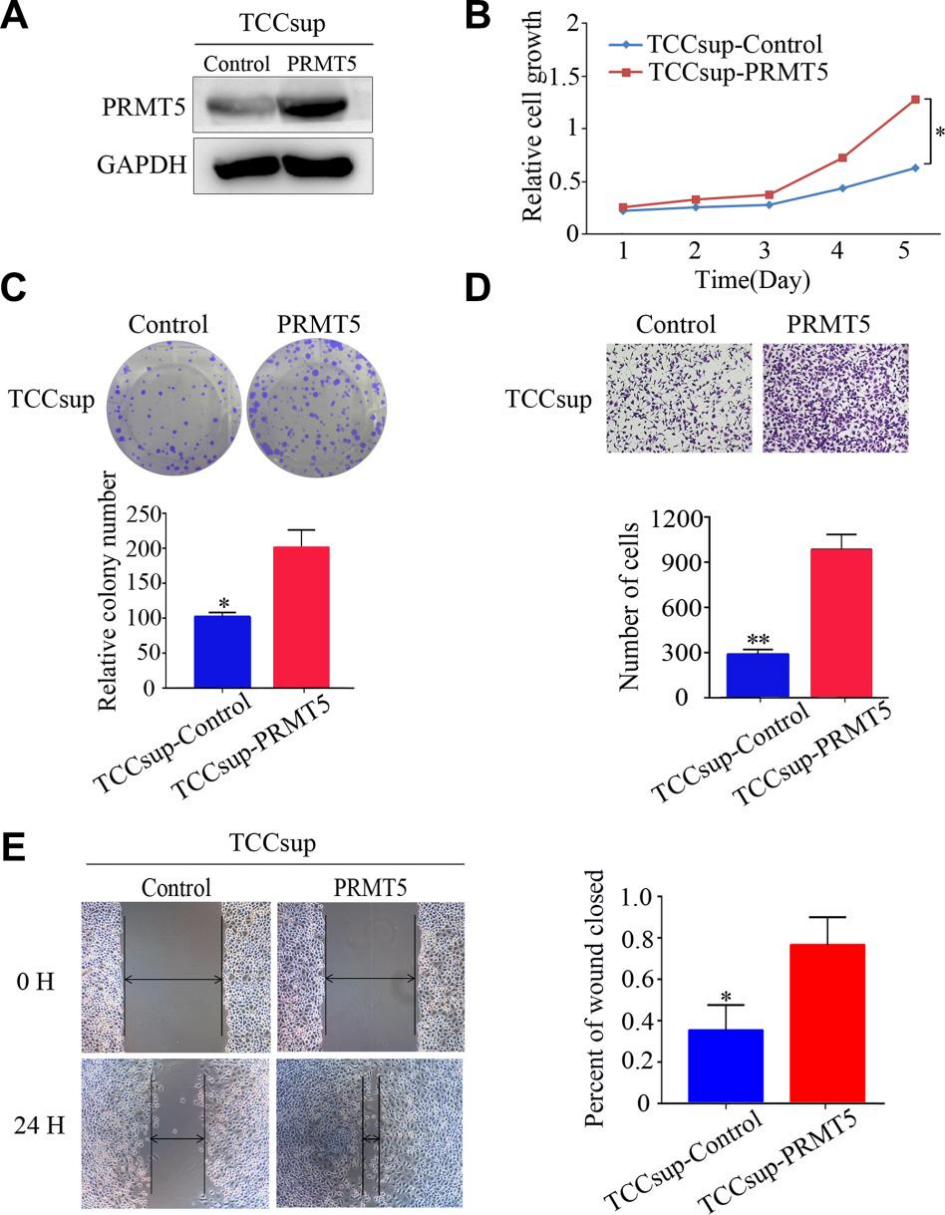
**Upregulated expression of PRMT5 promoted the proliferation and aggressiveness of bladder cancer cells.** (**A**) Western blotting showed successful overexpression of PRMT5 in TCCsup cells. (**B**, **C**) Cell proliferation was promoted by the CCK-8 assay and the colony formation assay. (**D**, **E**) Cell invasion and migration of bladder cancer cells were detected using the transwell assay and the wound-healing assay. Error bars show the standard error of the mean. *P < 0.05, **P < 0.01.

We investigated the function of PRMT5 in BUC cells in vitro using western blotting and confirmed that the relative level of PRMT5 expression was downregulated in Biu87 and T24 cells by two specific siRNAs compared with that in the negative control group (Figure 3A). Cell proliferation was inhibited in cells with knockdown of PRMT5 as a result of siRNA. EdU assay was applied to explore the function of PRMT5 in promoting cell growth. There were significantly more EdU-positive T24 or Biu87 cells in the negative group than in the si-PRMT5 group after transfection of the indicated siRNA (Figure 3B). Next, the cell growth assay using cell counting kit-8 revealed that PRMT5 knockdown significantly decreased the number of the two indicated BUC cell lines (*P* < 0.05, Figure 3C). In the colony formation assay, both T24-siRNA and Biu87-siRNA cells formed fewer and smaller colonies than the negative control cells (*P* < 0.05, Figure 3D). Similarly, gene silencing of PRMT5 also significantly reduced BUC cell invasion and migration abilities (*P* < 0.05, Figure 3E, 3F). When we upregulated PRMT5 in the TCCsup cells (Figure 4A), the transfected TCCsup-PRMT5 cells demonstrated a significantly higher proliferative capacity compared with the respective control cells (*P* < 0.05, Figure 4B, 4C). In addition, the transwell migration assay and the cell scratch assay, which were performed to evaluate the invasion and migration ability of the transfected TCCsup-PRMT5 cells, indicated that PRMT5 significantly enhanced the migration capacity of BUC cells in vitro (*P* < 0.05, Figure 4D, 4E). These results suggest that PRMT5 promotes cell proliferation, invasion, and migration in BUC in vitro.

**Figure 5:**
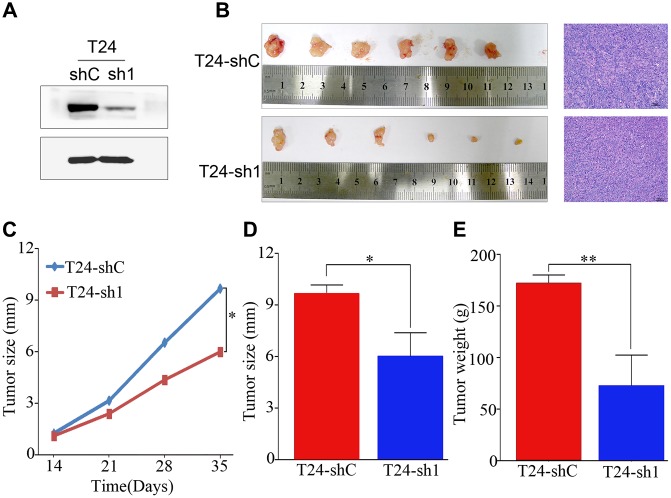
**PRMT5 had a strong oncogenic function in bladder cancer.** (**A**) Western blotting showed that PRMT5 was efficiently knocked down by PRMT5-shRNA. (**B**) Representative images of the tumor xenografts inoculated with T24-shC and T24-sh1 cells and hematoxylin and eosin staining of tumor xenografts are shown. (**C–E**) Growth curve and histogram analysis of the size and weight of xenograft tumors. Error bars show the standard error of the mean. sh, short hairpin RNA; T24-shC: T24-shRNA/negative control; T24-sh1: T24-shRNA. *P < 0.05, **P < 0.01.

To confirm the effect of PRMT5 on tumor growth in vivo, a nude mouse subcutaneous xenograft tumor model was established. Western blotting identified the shRNA efficiency of PRMT5 downregulation at the protein level (Figure 5A). As shown in Figure 5B, the subcutaneous xenografts in mice inoculated with T24-sh1 cells were smaller than those in the control group. This result was confirmed using hematoxylin and eosin staining. The weight and size of the tumors were significantly reduced in the T24-shRNA group compared with the negative control group (*P* < 0.05, Figure 5C–5E).

### PRMT5 affects cell cycle G1/S arrest and mTOR signaling pathway activity

**Figure 6:**
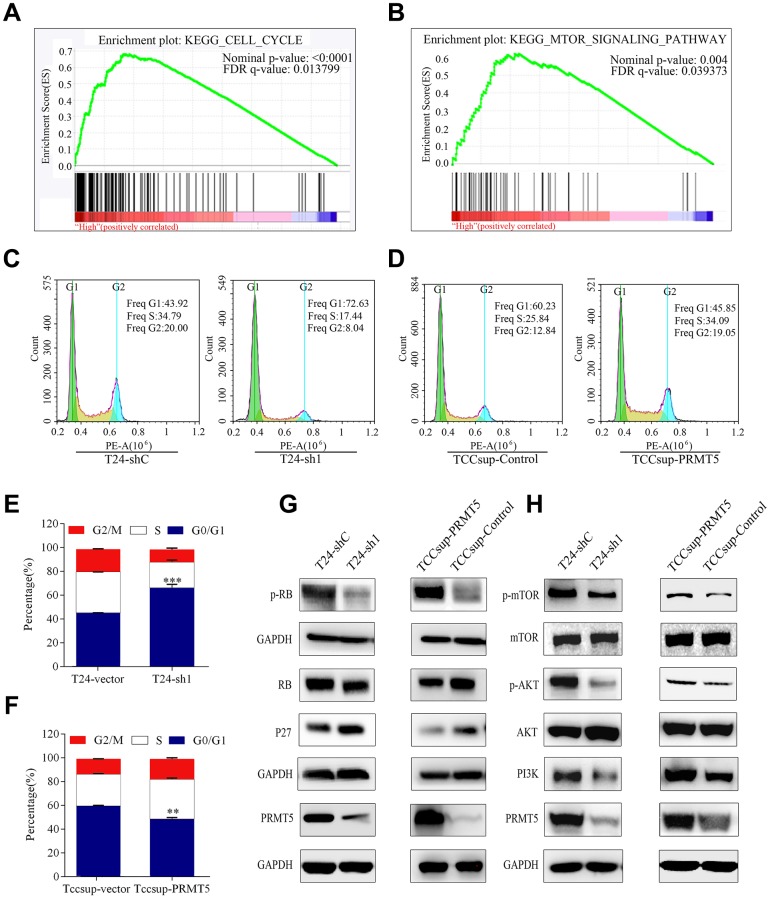
**High PRMT5 expression in bladder cancer promoted tumor progression through the cell cycle and the mTOR signaling pathway.** (**A**, **B**) GSEA indicated that high or low PRMT5 expression in bladder cancer is correlated with the cell cycle and the mTOR signaling pathway. Values were row-scaled to show the relative expression. (**C**) Flow cytometric analysis of the cell cycle showed that PRMT5 knockdown caused G1 cell cycle arrest. (**D**) PRMT5 facilitated the G1 to S phase transition of the cell cycle in bladder cancer. Corresponding statistics of knockdown (**E**) and overexpression (**F**) are presented by a bar graph (*P < 0.05, **P < 0.01, ***P < 0.001). (**G**, **H**) Western blots were performed after knockdown or overexpression of PRMT5 to evaluate the relevant protein expression of the cell cycle and the mTOR signaling pathway.

To investigate the biological pathway of PRMT5 in relation to BUC pathogenesis, gene set enrichment analysis (GSEA) was conducted using GSE133624. GSEA enrichment plots show a significant positive correlation between cell cycle and mTOR signaling pathway and PRMT5 in BUC (Figure 6A, 6B). To confirm that PRMT5 promotes cell proliferation by regulating the cell cycle, flow cytometry was performed to determine the effect of PRMT5 on cell cycle G1/S arrest. The proportion of cells in the G0/G1 phase significantly decreased after treatment with PRMT5 knockdown. This arrest corresponded with the observed result of proliferation and self-renewal, as shown in Figure 3. Furthermore, the G0/G1 phase increased in response to PRMT5 overexpression in BUC cells (*P* < 0.01, Figure 6C–6F).

Hypophosphorylated retinoblastoma (Rb) protein is a crucial regulator of cell transit from the G1 to S phase [18–20]. We investigated the phosphorylation status of Rb in the depletion and overexpression of PRMT5. The decreased level of phosphorylated Rb in BUC was consistent with the observation of PRMT5 depletion, suggesting that PRMT5 affects the cell cycle in BUC by controlling Rb activation (Figure 6G). p27 inhibits cyclin E activity, which causes hyperphosphorylation of Rb proteins [21–23]. Therefore, we examined whether PRMT5 depletion increased the expression of p27 in BUC via western blot analysis (Figure 6G).

Subsequently, we tested the impact of PRMT5 on the PI3K/Akt/mTOR pathway in BUC cells. Western blot analysis demonstrated that PRMT5 knockdown led to downregulation of PI3K, which deactivated Akt and mTOR phosphorylation. Conversely, PRMT5 upregulation promoted PI3K, resulting in activation of Akt and mTOR phosphorylation (Figure 6H)

## DISCUSSION

BUC is the seventh most common cancer in men worldwide. The TNM staging system is based on the extent of disease at presentation and clinicopathology and provides clinical prognostic indicators for BUC. However, patients with the same TNM stage and undergoing the same treatment display considerable variability in recurrence and survival [24]. Many BUC patients experience tumor recurrence and metastasis. Determination of a patient’s prognosis using molecular biomarkers before treatment may allow more effective use of adjuvant therapy, thus improving patients’ quality of life [25, 26]. Many novel oncogenes, such as chromosome 14 open reading frame 166 [27], trimethylation of lysine 27 on histone H3 [28], and maelstrom [5], promote malignant phenotypes in BUC. However, they are not precise enough to predict the patient’s prognosis.

Many arginine and lysine methyltransferases have been reported in cancers. Arginine methylation is an important regulator of biological function, tumorigenesis, and tumor progression [11, 14]. PRMT5 is the predominant type II methyltransferase and has a variety of functions. Together, these functions may contribute to increased tumorigenesis, leading to malignancies such as prostate, hepatocellular, and breast cancer and melanoma [12, 14]. PRMT5 and its substrate-binding partner WDR77 regulate alternative splicing through methylation of ZNF326 in breast cancer [17]. Upregulation of PRMT5 in glioblastoma cells increases their self-renewal capacity and proliferation through the PRMT5–PTEN molecular pathway [29]. These findings indicate that high PRMT5 expression promotes the proliferative and migratory processes of several types of solid cancer in humans. However, the role of PRMT5 in BUC oncogenesis had not been previously clarified. Thus, in this study, we explored the potential oncogenic role of PRMT5 in BUC. First, we observed that PRMT5 was significantly upregulated in BUC tissue compared with adjacent nontumor tissue. Recent reports have indicated that overexpression of the PRMT5 protein is positively correlated with advanced disease stage and adverse prognosis of certain human solid tumors [30, 31]. In patients with BUC, we also found that elevated PRMT5 was correlated with poor disease prognosis. As shown by Kaplan-Meier analysis and multivariate Cox analysis, PRMT5 was an independent adverse prognostic factor for overall and disease-free survival in patients with BUC. These findings indicate that PRMT5 may promote the growth and progression of BUC.

PRMT5 is a ubiquitous and evolutionarily conserved protein, but it may be involved in proliferation, migration, and invasion of cancer. PRMT5 upregulation results in increased proliferation and anchorage-independent colony growth, whereas cellular proliferation and colony formation in cancer are significantly inhibited by PRMT5 knockdown [14, 32–34]. Studies have considered direct inhibition resulting from increased translation [34, 35]. These reports indicated that there was one mechanism by which PRMT5 controls cell growth. Based on the upregulation of PRMT5 in BUC in our tests, we conducted phenotypic experiments and explored the potential biological role of PRMT5 in vivo and in vitro. In our experiments, PRMT5 knockdown resulted in significant inhibition of growth, migration, and invasion of BUC cells. Clearly, further study is needed to more accurately detect the signaling pathway involved in the oncogenic process of BUC induced by the upregulation of PRMT5. We conducted bioinformatics analysis, and the array showed that expression of PRMT5 in BUC was significantly associated with the cell cycle and the mTOR signaling pathway.

Distorted activity of proteins that affect the cell cycle induces abnormal proliferation, resulting in malignant transformation. We demonstrated that PRMT5 facilitated the progression of cells through the G1 to S phase, in accordance with the bioinformatics analysis. PRMT5 mediated the repression of p27 and the activation of Rb in BUC. Furthermore, our data showed that PRMT5 boosted the activity of the mTOR signaling pathway in BUC through upregulation of PI3K and Akt. Specifically, PRMT5 inhibition markedly impaired the PI3K/Akt/ mTOR pathway. PRMT5 may affect BUC tumorigenesis through this pathway. However, the relationship between PRMT5 and the PI3K/Akt/mTOR signaling pathway needs further research.

In summary, we demonstrated that PRMT5 upregulation in BUC is correlated with a poor prognosis and BUC proliferation, migration, and invasion. In addition, PRMT5 affects BUC tumor progression by distorting the Rb and p27 proteins involved in the cell cycle and facilitating the PI3K/Akt/mTOR signaling pathway. PRMT5 inhibitors may be a potential therapeutic approach that offers promising perspectives of BUC in the future.

## MATERIALS AND METHODS

### Cell lines and cell culture

J82, UMUC, RT4, Biu87, TCCsup, 5637, and T24 bladder cancer cells from the American Type Culture Collection were cultured in RPMI 1640 medium or DMEM (Invitrogen, Carlsbad, CA, USA) with 10% fetal bovine serum (South Logan, UT, USA). All of cells were cultivated in an atmosphere at 37°C with 5% CO_2_.

### qRT-PCR

RNA isolated with Trizol (Invitrogen) from the samples was quantified using an ultramicrospectrophotometer (NanoDrop ND-1000, USA), and it was reverse transcribed into cDNA using PrimeScriptTM Master Mix (TaKaRa, Japan). Then qRT-PCR was run on a Light-Cycler 480 instrument (Roche Diagnostics, Germany). The relative quantitative value was calculated using the 2-ΔΔCt method. The final result was recorded as fold change. Every sample was performed with three replicates.

### Western blot and immunohistochemistry

All protein samples were extracted from the clinical bladder tissues and BUC cell lines using RIPA buffer (P00013C, Beyotime) and proteinase inhibitor cocktail (Roche, USA). The concentration of the protein was quantified using the BCA protein assay (Thermo Fisher Scientific, USA). Western blots were implemented in accordance with the standard methods [36]. Immunohistochemistry, which was used to determine the expression of PRMT5 in BUC tissues, has been described in previous studies [27]. An anti-PRMT5 antibody (1:500; Proteintech Group) was used.

### RNA interference

To knock down the expression of PRMT5, two PRMT5 small-interfering RNAs (siRNAs) and the cognate control siRNA were synthesized by Gene Pharma (Shanghai, China). Then, 20 μm of siRNA was transfected into the indicated cells in six-well plates through the Lipofectamine 2000 Reagent (Invitrogen) according to the manufacturer’s instructions. The target sequences of PRMT5 for constructing the siRNAs are as follows: siRNA1, 5′-CCAGUUUGAGAUGCCUUAUT TAUAAGGCAUCUCAAACUGGGC-3′; siRNA2, 5′-GUUUCAAGAGGGAGUUCAUTTAUGAACUCCCUCUUGAAACGC-3′. Western blot was used to verify the effect of gene silencing 48 hours after transfection.

### Cell counting kit-8 (CCK-8) assay

The indicated cells were sowed in each well at a density of 2,000 cells on 96-well plates. We examined the cell growth every 24 hours with CCK-8 (Beyotime Technology, China) after incubation for 30 minutes at 37°C. According to the reference wavelength, the absorbance value was detected at 450 nm.

### Colony formation assay

The indicated cells were sowed in each well at a density of 500 cells on six-well plates. After cultivation for 7 days, the colonies were stained with crystal violet and visualized with a microscope.

### 5-Ethynyl-2'-deoxyuridine (EdU) assay

5-Ethynyl-20-deoxyuridine (EdU), a more specific and sensitive method, was mainly used as the marker of cellular replication activity [37]. For an EdU incorporation assay, cells were placed on every confocal well (2,000 cells per well) and cultured for 24 hours. EdU fluorescent staining was performed using a commercial cell-light EdU Apollo 567 in vitro kit (RIB&BIO, Guangzhou, China) according to the manufacturer’s protocol. A fluorescence confocal microscope (Nikon, Japan) was used to directly detect staining for PRMT5 in the EdU-labeled cells.

### Transwell invasion assay

Biu87 and T24 cells (5×10^4^ cells/well) were plated on the upper chamber of 24-well plates in RPMI 1640 without serum. Then 20% formaldehyde was used to fix the invading cells, and crystal violet was used for staining at 24 hours after seeding.

### Cell wound-healing assay

The cell monolayer in six-well plates was scraped by a sterile 100-mL pipette tip and then washed with PBS. The scratched wells were cultivated in a serum-free medium. At the time points of 0 and 24 hours, the scratched cell images were captured to analyze migration by an inverted microscope.

### Transfection of the recombinant lentiviral vector

Stocks of virus were produced in 293-t cells, as described in previous studies [38]. The retroviruses carrying PRMT5 short hairpin RNA (PRMT5-shRNA) were transfected into T24 cells, whereas the lentiviral vectors with PRMT5 were transfected into TCCsup cells. Homologous and empty lentiviral vectors were transfected into cancer cells as controls. Puromycin (Santa Cruz Biotechnology, USA) was used to select stable cells.

### Tumor growth in xenografts

Twelve male BALB/c nude mice were divided into two groups. They were fed with sterilized feed and pure water in a specified pathogen-free environment with air laminar flow chamber. Feed, bedding, and cages for indoor use were autoclaved and transported through a sterile inlet chamber to exclude any microorganisms. Then mice were divided into two groups and inoculated subcutaneously with 1×10^7^ of T24-shRNA or T24-negative control cells. The xenografts were observed and sized using Vernier calipers every week after injection. Five weeks later, all of the mice were euthanized by cervical dislocation, and the subcutaneous tumor xenografts in the mice were collected. The weight and maximum diameter of the tumor xenografts were measured using an electronic scale and Vernier calipers, respectively. Finally, the tumor xenografts were fixed in 10% formalin and embedded in paraffin for hematoxylin and eosin staining and the histopathological analysis.

### Cell cycle analysis

Double thymidine was used for synchronization. Posttreatment cells were treated with the cell cycle analysis kit (4Abio, Beijing, China) according to the standard protocol. Stained cells were tested using a flow cytometer (cytoFLEX, Beckman, USA).

### Patient and specimen characteristics

PRMT5 expression data and detailed clinical information of BUC patients were first ascertained from the data sets of the Oncomine database (https://www.oncomine.org) and The Cancer Genome Atlas (https://www.cancer.gov/about-nci/organization/ccg/research/structural-genomics/tcga). One hundred thirty-two clinical BUC samples from Chinese patients from 2002 to 2016 were collected at the Sun Yat-Sen University Cancer Center. The clinical and pathological information collected included age, gender, surgery, tumor size, TNM stage, differentiation grade, vital status, and follow-up time, and tumors were classified according to the guidelines of the European Association of Urology [39]. All samples were from patients who had not yet received chemotherapy, radiotherapy, or immunotherapy and were obtained after surgery. All samples were acquired after receiving informed consent and approval from the Ethical Committee of Sun Yat-Sen University Cancer Center (Guangzhou, China).

### Statistical analysis

Using SPSS 20.0 (SPSS, Inc., Chicago, IL, USA) and GraphPad Prism 6.0 (GraphPad Software, La Jolla, CA, USA), the mRNA levels of PRMT5 in BUC tumor tissues were compared with those in adjacent nontumor tissues using a paired student’s *t* test. Relationships between PRMT5 expression and clinical factors were analyzed. Descriptive statistics and the χ^2^ test were used to assess the differences between groups. Overall survival was analyzed by the Kaplan-Meier method using the log-rank test. Univariate and multivariate Cox regression analyses were performed to assess the correlation among age, gender, tumor size, tumor stage, and differentiation grade. *P* values *<* 0.05 were considered statistically significant.

### Ethics approval

The Ethical Committee of Sun Yat-Sen University Cancer Center provided permission to use the clinical samples. All patients provided written consent for the clinical samples and data to be used for scientific research. The animal experiment was approved by the Animal Ethical and Welfare Committee of Sun Yat-Sen University and abided by the Guide for the Care and Use of Laboratory Animals.
